# Evaluating dried salted cod amino acid signature for nutritional quality assessment and discriminant analysis

**DOI:** 10.3389/fnut.2023.1144713

**Published:** 2023-04-14

**Authors:** M. A. G. Quaresma, G. Pereira, M. L. Nunes, C. Sponda, A. Jardim, H. Gonçalves, C. Santos, L. C. Roseiro

**Affiliations:** ^1^CIISA—Centre for Interdisciplinary Research in Animal Health, Faculty of Veterinary Medicine, University of Lisbon, Lisbon, Portugal; ^2^AL4AnimalS—Associate Laboratory for Animal and Veterinary Sciences, Lisbon, Portugal; ^3^CIIMAR, Interdisciplinary Centre of Marine and Environmental Research, Terminal de Cruzeiros do Porto de Leixões, University of Porto, Matosinhos, Portugal; ^4^Food and Veterinary Department of Setúbal, General Directorate of Food and Veterinary (DGAV), Setúbal, Portugal; ^5^Food Technology and Safety Division, National Institute for Agricultural and Veterinary Research (INIAV, IP), Quinta do Marquês, Oeiras, Portugal

**Keywords:** *Gadus morhua*, *Gadus macrocephalus*, dried salted cod, total protein content, amino acid profile, canonial discriminant analysis

## Abstract

**Aim:**

Thus, the aim of this study was to answer three scientific questions: (1) Are the protein content and amino acid profile of dried salted cod influenced by species (Gadus morhua and Gadus macrocephalus)? (2) Are the protein content and amino acid profile of dried salted cod influenced by the geographical area of capture (Iceland and Norway)? and (3) Does the amino acid profile have the potential to be used as a discriminator of species and geographical areas of capture?

**Methods:**

A total of 45 dried salted cods (2–3 kg of dry weight; *n* = 15 samples/origin) were used in this study. The Atlantic cod was fished in the Atlantic northeast (FAO 27 area) within the Exclusive Economic zones (EEZ) of Norway (*n* = 15) and Iceland (*n* = 15), while the Pacific cod was caught in the Pacific northeast (FAO 67 area) within the Alaska EEZ (*n* = 15). Total protein content was determined by the Kjeldahl method, in accordance with the AOAC procedures. The amino acid profile was analyzed by HPLC with fluorescence detection (at excitation and emission wavelengths of 338 and 425 nm, respectively).

**Results:**

The Atlantic cod presented higher contents of total protein (33.90 versus 33.10 g/100 g of cod edible portion; *p* = 0.017) and total amino acid contents (32.52 versus 32.04 g/100 g of cod edible portion; *p* = 0.015) but displayed lower percentage of indispensable amino acids (32.16 versus 32.83 g/100 g of protein; *p* < 0.001) than Pacific cod. Among the Atlantic cod harvesting locations, the Norwegian cod displayed higher total amino acid contents (96.91 versus 96.81 g/100 g of protein; *p* = 0.012) and higher percentage of indispensable amino acids (35.38 versus 28.94 g/100 g of protein; *p* = 0.042) than the Icelandic counterpart. A correct classification of 100% was obtained for the Pacific and Icelandic cod varieties, but the classification accuracy in the Norwegian cod was of just 86.67%, since 2 samples out of 15 were incorrectly classified as Icelandic.

**Conclusion:**

The comparison of cod species showed that the Atlantic cod had a significantly lower EAAI than the Pacific cod (*p* < 0.001; 88.23 versus 88.61). On the other hand, the comparison of the two origins in the Atlantic cod, showed that Norwegian cod displayed a significantly higher EAAI than the Icelandic cod (99.15 versus 77.32). The assessment of the EAAI allows the classification of the protein’s nutritional quality, allowing us to classify both cod species as a good protein source to human diet. However, within the Atlantic cod, the Norwegian cod’s protein is classified as high quality, while the Icelandic cod attain the classification of useful quality. Regarding the amino acid profile discriminatory potential to classify cod samples. The results show that the AA profile has 100% accuracy in the separation of cod species, but was not globally efficient in the differentiation of the Norwegian from the Icelandic cod.

## 1. Introduction

Globally, fishery and aquaculture products account for 17% of the total animal protein consumed by the human population (1). Fish is a source of high-quality protein, low in fat but high in *n*–3 PUFA, as eicosapentaenoic and docosahexaenoic acids. Fish is also an important source of several vitamins (B1, B3, B6, B12, and D) and minerals (including calcium, phosphorus, iron, selenium, zinc, iodine, magnesium, and potassium). The consumption of fisheries and aquaculture products in the European Union (EU-28) achieve 24.2 kg/inhabitant/year (2).

The Atlantic cod (*Gadus morhua*) has been among the ten most landed species worldwide between 1950 and 2018 (1), and it is still the third most consumed fish species in the European Union, after tuna and salmon, being associated with 9% share of total seafood consumption, with a *per capita* consumption of 2.1 kg (2). Regarding the annual *per capita* consumption of cod, the average consumption of the Portuguese population (7 kg of dried salted cod inhabitant/year, which is equivalent to 20 kg of fresh cod inhabitant/year), far exceeds the average consumption of the European population, estimated in 1.7 kg (2). If a single food could represent a country, Portugal would be historically and culturally typified by dried salted cod (bacalhau), which has been an important food resource for the Portuguese population since the 16th century, and used for the development of hundreds of traditional recipes throughout the country (3).

The consumption of dried salted cod persists until nowadays, being primarily consumed in some Mediterranean countries (Portugal, Spain and Italy) and in the Latin America (4). During cod salting and ripening periods, several changes occur in the edible portion, at both flavor and textural levels, which persist over cooking (5). Dried salted cod flavor results from a complex combination of enzymatic and/or chemical reactions such as lipid oxidation, Maillard and Strecker degradation reactions (5, 6), which impart the formation of numerous volatile compounds with odorous properties (7). Moreover, salting improves flavor because decreases water activity, which can lead to an effective increase in the concentration of flavory components and improve their volatility (8).

Atlantic cod stocks have been considered overexploited and lost 3–49% of their total biomass since 1970 (9). Therefore, this species was listed as ‘vulnerable’ by the International Union for the Conservation of Nature (IUCN), a condition that persists until nowadays (10), being currently protected by strict management. Even so, data from FAO revealed that 1.13 million tons of Atlantic cod and 0.43 million tons of Pacific cod (*Gadus macrocephalus*) were landed in 2019, denoting that the Atlantic cod (72.3% of the total load recorded for cod) is by far the most captured of these two species (11).

Considering the genetic and phenotypic similarities between the Atlantic and Pacific cod species (12, 13), it would seem reasonable to transfer part of the fishing pressure from the Atlantic cod towards the Pacific counterpart for a period of time established in accordance with the Pacific cod biomass surveillance data. Anticipating such expectable change in cod’s trade, this study aimed to expand the knowledge on the composition of dried salted cod protein obtained from the Atlantic and Pacific cod species. Therefore the study was supported by three scientific questions: (1) Are the protein content and amino acid profile of dried salted cod influenced by cod’s species (*Gadus morhua* and *Gadus macrocephalus*)?; (2) Are the protein content and amino acid profile of dried salted cod influenced by the geographical area of capture (Iceland and Norway)? and (3) Does the amino acid profile possess discriminate potential between species and geographical harvesting areas?

## 2. Materials and methods

### 2.1. Sample characterization and preparation

A total of 45 dried salted cods (weighting 2–3 kg of dry weight/specimen; *n* = 15 samples/origin) were used in this study. The Atlantic cod was caught off the Atlantic northeast (FAO 27 area) within the Exclusive Economic zones (EEZ) of Norway (*n* = 15) and Iceland (*n* = 15), while the Pacific cod was caught in the Pacific northeast (FAO 67 area) within the Alaska EEZ (*n* = 15). The dried salted cod specimens used herein were randomly selected, among those sharing analogous fishing and processing methodologies, and were all collected at Riberalves company (Carvalhal, Torres Vedras, and Portugal). Dried salted cod used herein shared equal salting and drying procedures. According to Good Manufacturing Practices of Riberalves, the average time between cod harvesting and the salting and drying processes was two to 3 months. Salting and drying processes were performed according to commercial procedures for specimens weighing 2–3 kg at the end of the process.

The dried salted cod samples used in the analyses were obtained from the central portion of cod’s loin, the skin and bones were manually removed, and the remaining part of muscle tissue (designated hereafter as edible portion) was cut in thin slices before blending it in a food processor (Moulinex, France). The homogenized material was vacuum packed and frozen/stored at − 20°C until subsequent analysis. In all assays, the 45 samples were analyzed in duplicate, and the results were only accepted when the coefficient of variation between duplicates was below 5%, for both the total protein and total amino acid contents.

### 2.2. Reagents

General pro-analysis grade chemicals (hydrochloric acid, sodium acetate, sodium tetraborate, 2-mercaptoethanol) were purchased from Merck Biosciences (Darmstadt, Germany); ortho-phthalaldehyde, methanol and tetrahydrofuran, all HPLC-grade, were supplied by Sigma Aldrich (St. Louis, United States) and Milli Q water was HPLC-grade. The amino acids standards (aspartic acid, asparagine, glutamic acid, glutamine, serine, histidine, glycine, threonine, arginine, alanine, tyrosine, valine, methionine, tryptophan, phenylalanine, isoleucine, leucine and lysine) were supplied by Sigma Aldrich (St. Louis, United States).

### 2.3. Protein and amino acid (AA) analysis

Total protein content in dried salted cod edible portion was determined by the Kjeldahl method, in accordance with the Association of Official Analytical Chemists procedures (14).

The AA composition of edible portion was analyzed according to the protocol previously described (15), with minor modifications, previously described in detail (16).

Fluorescence was monitored at excitation and emission wavelengths of 338 and 425 nm, respectively. The AA were identified by comparison with the retention time of standards and their quantification was based on the external standard technique, from a standard curve of peak area vs. concentration. The detailed amino acid profile is expressed as g/100 g of protein, while the amino acid partial sums are expressed either as g/100 g of protein and as g/100 g of dried salted cod edible portion.

The amino acid flavor properties, i.e., “sweet,” “bitter,” “acidic (sour)” and “umami (delicious)” were established according to studies previously published (17–19):

(1) Sweet is the sum of alanine, glycine, hydroxyproline, proline, serine and threonine;
(2) Bitter is the sum of arginine, histidine, isoleucine, leucine, methionine, phenylalanine, tryptophan and valine;
(3) Acidic is the sum of asparagine, aspartic acid, glutamic acid and histidine; and
(4) Umami is the sum of aspartic and glutamic acids.

### 2.4. Protein quality ratios and indices

Cod’s protein nutritional quality was assessed considering the amino acid score (AAS) and the essential amino acid index (EAAI). The AAS was calculated using the reference scoring pattern of the adult (isoleucine (Ile): 3, leucine (Leu): 6.1, lysine (Lys): 4.8, Methionine + cysteine (Met + Cys): 2.3, phenylalanine+ L-tyrosine (Phe + Tyr): 4.1, threonine (Thr): 2.5, threonine (Trp): 0.67, valine (Val): 4.0, and histidine (His): 1.6 expressed in g/100 g of protein) (20). The EAAI was calculated as the geometric mean of the ratios of the essential amino acid contents in cod protein (His, Ile, Leu, Lys, Met, Thr, Trp, Val and Phe + Tyr; expressed as g/100 g of protein), relative to their respective amounts in the amino acid scoring pattern (21), *n* = 9 (the number of EAA) and was expressed in percentage. The AAS and the EAAI in cod’s edible portion were calculated according to the equations (22).


$$\mathrm{AAS}=\frac{\mathrm{mg}\;\mathrm{of amino acid in}\;1\;g\;\mathrm{of test protein}\;}{\mathrm{mg}\;\mathrm{of amino acid in reference pattern}}*100$$

$$\mathrm{EAAI}=100*\sqrt[{n}]{\frac{\mathrm{Aa}1_{c}}{\mathrm{Aa}1_{\mathrm{rp}}}*\frac{\mathrm{Aa}2_{c}}{\mathrm{Aa}2_{\mathrm{rp}}}*\ldots *\frac{\mathrm{Aa}n_{c}}{\mathrm{Aa}n_{\mathrm{rp}.}}}$$

Where Aa refers to amino acids, the subscript “c” to the protein sample in analysis, “rp” to the reference pattern, and “n” to the number of essential amino acids.

### 2.5. Statistical analysis

Throughout the results and discussion, the term superiority (expressed as %) was calculated as (maximum value – minimum value)/minimum value.

**Figure 1:**
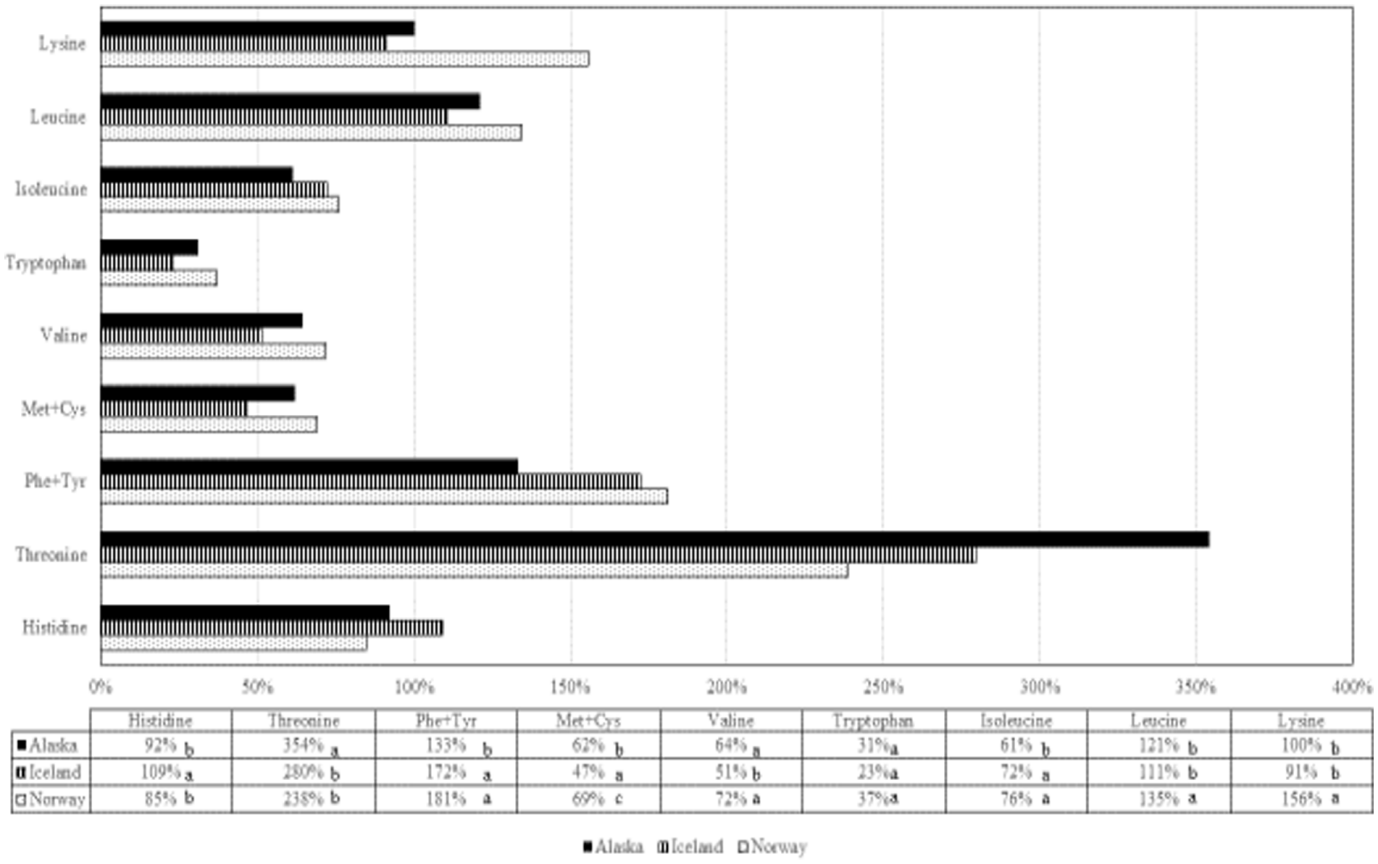
Amino acid score (AAS) for adults according to standards reported by the FAO/WHO for adults.

The statistical analysis was accomplished using several statistical tests using SAS software (SAS Inst., Cary, NC, United States; version 9.4), namely: Proc. MIXED, Proc GLM and the Canonical discriminant analysis (CDA). The Proc. MIXED procedure of was applied, considering the species plus origin as single effect. A total of 2 orthogonal contrasts were used to evaluate the species effect (Atlantic cod versus Pacific cod; A vs. P) and the origin effect within Atlantic cod (Norwegian versus Icelandic cod; N vs. I). The values presented in Tables 1–3 are the least squares means and the standard error of the mean (SEM). The Proc. GLM was applied in a single analysis (Figure 1), considering the cod’s origin as a single effect. The significance was established at *p* < 0.05, and whenever a significant difference was detected, the least squares means were compared for alpha = 0.05, using the LSD test adjusted by the Tukey method.

**Table 1:** Total protein (TP) and total amino acid (TAA) contents, amino acid partial sums (TAA, IAA, CIAA and DAA), amino acid ratios, and sums of amino acids associated with flavor in the Atlantic and Pacific cod.

	Atlantic cod		Pacific cod	SEM	Contrasts	
	Norway	Iceland	Alaska		A vs P	N vs I
TP^1^	31.95	35.85	33.10	1.17	0.017	0.324
TAA^2^	30.32	34.71	32.04	1.13	0.015	0.288
Partial sums (g/100 g of protein)						
Σ TAA^3^	96.91	96.81	96.61	0.11	<0.021	0.012
Σ IAA^4^	35.38	28.9	32.83	0.76	<0.001	0.042
Σ CIAA^5^	25.58	29.19	27.04	0.92	0.011	0.207
Σ DAA^6^	36.05	38.67	36.74	0.69	0.009	0.423
Ratios						
IAA/TAA	0.37	0.30	0.34	0.008	<0.001	0.036
DAA/TAA	0.37	0.40	0.38	0.007	0.013	0.470
CIAA/TAA	0.26	0.30	0.28	0.01	0.013	0.222
IAA/DAA	0.98	0.75	0.89	0.03	<0.001	0.066
Flavour (g/100 g edible portion)						
Bitter	10.40 ± 0.42	12.00	10.05	0.42	0.001	0.558
Sweet	8.88 ± 0.50	10.79	11.74	0.50	0.437	<0.001
Acidic (Sour)	7.60 ± 0.27	9.12	7.98	0.27	<0.001	0.324
Umami	7.17 ± 0.24	8.49	7.49	0.24	<0.001	0.358

1 TP: total protein (expressed as g/100 g edible portion);

2 TAA: total amino acids (expressed as g/100 g edible portion);

3 TAA: total amino acids (expressed as g/100 g of protein);

4 IAA: indispensable amino acids (expressed as g/100 g of protein);

5 CIAA: conditionally indispensable amino acids (expressed as g/100 g of protein);

6 DAA: dispensable amino acids (expressed as g/100 g of protein);

Contrast A vs P: Atlantic cod versus Pacific cod;

Contrast N vs I: Norwegian cod versus Icelandic cod.

**Table 2:** Amino acid profile and essential amino acid index (EAAI) of Atlantic and Pacific cod edible portion.

	Atlantic cod		Pacific cod	SEM	Contrasts	
	Norway	Iceland	Alaska	A vs P	N vs I
Indispensable amino acids (IAA; expressed as g/100 g edible portion)						
Histidine	0.43	0.63	0.49	0.03	<0.001	0.196
Isoleucine	0.72	0.74	0.61	0.03	0.009	0.018
Leucine	2.58	2.43	2.43	0.12	0.600	0.394
Lysine	2.33	1.58	1.61	0.22	0.156	0.024
Methionine	0.50	0.38	0.47	0.02	<0.001	0.263
Phenylalanine	0.90	0.81	0.80	0.04	0.329	0.060
Threonine	1.89	2.52	2.96	0.18	0.673	<0.001
Tryptophan	0.76	0.53	0.65	0.14	0.306	0.568
Valine	0.90	0.73	0.85	0.04	0.002	0.260
Σ IAA	11.01	10.39	10.88	0.45	0.318	0.833
Conditionally indispensable amino acids (CIAA; expressed as g/100 edible portion)						
Arginine	3.60	5.71	3.74	0.33	<0.001	0.771
Glycine	2.47	2.49	3.49	0.18	0.028	<0.001
Proline	0.50	0.55	0.81	0.05	0.075	<0.001
Tyrosine	1.43	1.72	1.01	0.12	0.002	0.023
Σ CIAA	8.01	1	9.05	0.48	0.002	0.132
Dispensable amino acids (DAA; expressed as g/100 g edible portion)						
Alanine	1.87	2.82	1.68	0.14	<0.001	0.342
Aspartic acid	2.96	3.79	2.98	0.12	<0.001	0.917
Glutamic acid	4.21	4.70	4.51	0.14	0.055	0.144
Hydroxyproline	0.70	0.60	1.18	0.10	0.010	0.002
Ornithine	0.12	0.12	0.16	0.002	<0.001	<0.001
Serine	1.44	1.81	1.61	0.07	0.001	0.090
Σ DAA	11.30	13.84	12.11	0.43	<0.001	0.185
EAAI* (%)	99.15	77.32	88.61	2.02	<0.001	<0.001

Contrast A vs P: Atlantic cod versus Pacific cod;

Contrast N vs I: Norwegian cod versus Icelandic cod.

**Table 3:** The amino acid score (AAS) for all the indispensable amino acids (mean ± standard deviation).

	Atlantic cod		Pacific cod	SEM	Contrasts	
	Norway	Iceland	Alaska	A vs P	N vs I
Amino acid score (AAS)						
Histidine	84.67	109.2	92.34	3.71	<0.001	0.151
Isoleucine	76.17	72.15	61.40	2.05	0.188	<0.001
Leucine	134.6	110.8	120.9	4.21	0.002	0.027
Lysine	155.6	60.93	100.3	12.7	0.022	0.004
Methionine^1^	69.23	46.74	61.96	1.75	<0.001	0.005
Phenylalanine^2^	181.2	172.3	133.3	8.63	0.161	<0.001
Threonine	238.4	279.5	354.0	16.4	0.411	<0.001
Tryptophan	37.17	23.32	31.01	6.40	0.177	0.499
Valine	71.98	51.27	64.25	2.27	<0.001	0.021

1 Methionine + Cystine.

2 Phenylalanine + Tyrosine.

Contrast A vs P: Atlantic cod versus Pacific cod;

Contrast N vs I: Norwegian cod versus Icelandic cod.

Canonical discriminant analysis (CDA) was applied to amino acid profile in order to discriminate and predict cod’s origin. Variable selection for CDA was achieved using: (1) the significant variables defined after ANOVA, considering the origin as single effect (Proc GLM, SAS Inst., Cary, NC, United States; version 9.4); (2) an interactive forward stepwise analysis (SAS, Proc STEPDISC) selects the variables with a major discriminant capacity; (3) CDA and cross-validation were conducted using Proc DISCRIM from SAS.

## 3. Results

### 3.1. Total protein and amino acid contents

Table 1 presents the Atlantic and Pacific cod total protein (TP) and total amino acid (TAA) contents (expressed as g/100 g of edible portion), the TAA, total indispensable amino acids (IAA), total conditionally indispensable amino acids (CIAA) and total dispensable amino acids (DAA) contents (expressed as g/100 g of protein), the amino acid ratios (IAA/TAA, DAA/TAA, CIAA/TAA and IAA/DAA) and the amino acid contribution to flavor (“sweet,” “bitter,” “acidic (sour)” and “umami (delicious)”). Table 2 presents the amino acid profile and the Essential Amino Acid Index (EAAI) of Atlantic and Pacific cod edible portion.

The cod species significantly influenced the TP and TAA contents (*p* < 0.05), and the Atlantic cod presented higher total protein (33.90 versus 33.10 g/100 g of cod edible portion) and TAA (32.52 versus 32.04 g/100 g of cod edible portion) than Pacific cod. On the other hand, the harvesting location of the Atlantic cod had no significant influence on TP (*p* = 0.324) and TAA (*p* = 0.288) contents. Despite the absence of a significant statistical difference (*p* > 0.05) between the Norwegian and Icelandic cod on TP and TAA contents, the Icelandic cod presented a superiority of 10.5 and 14.5% on TP and TAA contents, respectively.

### 3.2. Amino acid profile

The analysis of cod’s protein fraction (Table 1) revealed that cod species had a significant influence on TAA (*p* = 0.021), total IAA (*p* < 0.001), total CIAA (*p* = 0.011) and total DAA (*p* = 0.009), while the influence of the harvesting location in the North Atlantic was limited to TAA (*p* = 0.012) and IAA (*p* = 0.042). Regarding cod’s protein composition, the Atlantic cod presented higher TAA (96.86 versus 96.61 g/100 g of protein), higher total CIAA (27.39 versus 27.04 g/100 g of protein), total DAA (37.31 versus 36.74 g/100 g of protein), but lower total IAA (32.16 versus 32.83 g/100 g of protein) than Pacific cod. Among the Atlantic cod harvesting locations, the Norwegian cod displayed higher TAA (96.91 versus 96.81 g/100 g of protein) and total IAA (35.38 versus 28.94 g/100 g of protein) than the Icelandic counterpart.

Cod species significantly influenced (*p* < 0.05) all the AA ratios (IAA/TAA, DAA/TAA, CIAA/TAA, and IAA/DAA), whereas the harvesting location of Atlantic cod significantly influenced the ratio IAA/TAA (*p* = 0.036), but had no significant influence (*p* > 0.05) on IAA/DAA, DAA/TAA and CIAA/TAA. Apropos, the Atlantic cod presented lower IAA/TAA and IAA/DAA ratios, and higher DAA/TAA and CIAA/TAA than Pacific cod. Therefore, the Pacific cod presented higher percentage of IAA, being for that reason regarded of higher nutritional quality. Among Atlantic harvesting locations, Norwegian cod presented higher IAA/TAA ratio than the Icelandic cod, being regarded as better nutritional quality.

The Atlantic cod edible portion presented significantly (*p* < 0.01) higher contents of two IAA (histidine and isoleucine), two CIAA (arginine and tyrosine) and three DAA (alanine, aspartic acid and serine), but presented significantly (*p* < 0.05) lower contents of two IAA (methionine and valine), one CIAA (glycine) and two DAA (hydroxyproline and ornithine) than the Pacific counterpart. Within the North Atlantic, the Norwegian cod edible portion holds significant (*p* < 0.05) higher contents of one IAA (isoleucine) and one DAA (hydroxyproline), but presented lower contents of threonine (IAA), proline and tyrosine (CIAA). No significant differences (p > 0.05) between harvest locations were observed for the remaining AA.

Considering the amino acid profile, the cod species were associated with significant differences (*p* < 0.05) in 12 out of 19 individual AA, and such AA were accountable for 57.8–62.1% of TAA in cod’s edible portion. Whereas, the harvesting location in the Atlantic North was linked to 8 significant differences (*p* < 0.05) out of 19 individual AA, and these AA were responsible for10.1–10.3–5 of TAA in cod’s edible portion. Nonetheless, the analysis of the AA profile showed that the 10 predominant AA in Norwegian and Icelandic cod protein were the same, despite having a different ranking order, enclosing 4 DAA (alanine, aspartic acid, glutamic acid and serine), 3 CIAA (arginine, glycine and tyrosine) and 3 IAA (leucine, lysine and threonine). The comparison of the Atlantic and Pacific cod species showed a single difference among the ten predominant AA, the Atlantic cod encloses tyrosine (4.5–4.8 g/100 g of protein) while the Pacific counterpart holds hydroxyproline (3.5 g/100 g of protein) rather than tyrosine.

The assessment of the amino acid score (AAS) for each IAA, depicted on Table 3 revealed that cod species has significantly influenced the AAS of histidine (*p* < 0.001), leucine (*p* = 0.002), lysine (*p* = 0.022), methionine plus cysteine (*p* < 0.001) and valine (*p* < 0.001). On the other hand, the harvesting location within the Atlantic cod has significantly influenced the AAS of isoleucine (*p* < 0.001), leucine (*p* 0.027), lysine (*p* = 0.004), methionine plus cysteine (*p* = 0.005), phenylalanine plus tyrosine (*p* < 0.001), threonine (*p* < 0.001) and valine (*p* = 0.021).

A different evaluation of the AAS, depicted on Figure 1, allowed the identification of limiting amino acids (LAA) in cod’s protein, the ones presenting a value below 100%.

Besides the nutritional requirements and physiologic functions, the amino acids contribute to food flavor, particularly the free amino acids, that have been associated with at least one of the five primary tastes [sweetness, sourness (acid), saltiness, bitterness, and umami (delicious)] (17, 18). Assuming that similar processing and ripening processes were followed, with comparable proteolysis in both cod species, the results show that Atlantic cod embrace higher concentration of AA implicated in the perception of umami, sour and bitter tastes (*p* < 0.05; a superiority of 4.5, 4.8 and 11.4%, correspondingly), and no significant (*p* = 0.140) difference was observed regarding the sweet taste component. The comparison of Atlantic cod from Norway and Iceland showed that the later presented higher concentration of AA implicated in the perception of the sweet component (a superiority of 21.5%; *p* < 0.001), suggesting that Icelandic cod has greater potential to express the sweetness, than the Norwegian counterpart. Differences in the AA profile observed between cod species and harvesting locations within Atlantic suggest differences in their potential to express the bitter, acidic, sweet and umami tastes.

### 3.3. Discriminatory ability of cod’ amino acid profile

**Figure 2:**
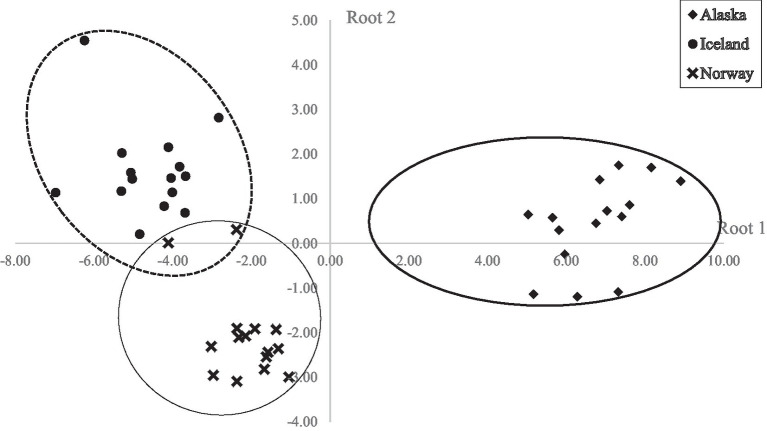
Plot of the discriminant functions (root 1 versus root 2) for the classification of cod’s origin.

Canonical discriminant analysis was applied to amino acid profile to assess their discriminant potential on cod’s origin. The results of canonical discriminant analysis, loadings of correlation matrix and discriminant functions are depicted in Table 4. A stepwise forward discriminant analysis was previously applied in order to select the most relevant variables for classification. In this procedure, variables that contribute with the highest discriminatory power were selected. The application of canonical discriminant analysis to selected variables produced two canonical discriminant functions, which maximized the ratio between class variance whilst minimizing the ratio within class variance. The coefficients obtained for each variable are presented in Table 4. A larger coefficient corresponds to a greater contribution of the respective variable to the discrimination between groups. For origin differentiation, the first two canonical discriminant functions were selected (Figure 2). The recognition ability of the discriminant model was evaluated by the correct classifications of 100% during the modelling step, allowing the differentiation of the three cod varieties. Afterwards, the prediction ability was carried out with a cross-validation method, in which one sample at a time was removed from the training set and considered as a test set. A correct classification of 100% was obtained for the Pacific and Icelandic cod varieties, but the classification accuracy in the Norwegian cod was of just 86.67%, since 2 samples out of 15 were incorrectly classified as Icelandic (Table 5).

**Table 4:** Results of the canonical discriminant analysis: loadings of correlation matrix between predictor variables (standardized canonical coefficients) and discriminant functions (roots 1 and 2), and some statistics for each function.

Variables	Root 1	Root 2
Alanine	−0.7983	1.1358
Arginine	−1.3714	0.4584
Isoleucine	−2.8901	0.2865
Ornitine	2.5972	1.3015
Proline	0.6766	−0.0029
Valine	1.1475	−1.2871
Total AA	2.2480	−0.1955
Statistics		
Canonical R	0.9782	0.8479
Eigenvalue	25.5199	2.5569
Cumulative proportion	0.9089	1.000
Probability	<0.001	<0.001

**Table 5:** Classification matrix of cross-validation for cod origin [number of observations and (percent classified into variety)].

Cod’s origin	Number of observations and (percentage classified into variety)			
	Alaska	Iceland	Norway	Total
Alaska	15 (100%)	0	0	15 (100%)
Iceland	0	15 (100%)	0	15 (100%)
Norway	0	2 (13.33%)	13 (86.67%)	15 (100%)
Total	15 (33.33%)	17 (37.78%)	13 (28.89%)	45 (100%)

Considering the data presented in Figure 2, presenting the discriminant roots 1 and 2. The Pacific cod caught in Alaska was located in the right half of the plot (embracing the positive values of root 1), most of the Pacific cod samples were located in the upper right quadrant (11 samples out of 15) embracing the positive values of root 2, but 4 samples were located in the right inferior quadrant, embracing the negative values of root 2. The Icelandic cod was totally located in the upper left quadrant, embracing the negative values of root 1 and the positive of root 2. On the other hand, the Norwegian cod was predominantly positioned on the left inferior quadrant (13 out of 15 samples), embracing the negative values of both roots, but one sample was placed on the top of the axis defined by root 1 and another sample was located in the left upper quadrant, and these two samples have been previously incorrectly classified by the cross-validation method.

## 4. Discussion

### 4.1. Total protein and amino acid contents

The protein content in cod’s edible portion is mutable and dependent of several variables such as age, sex, feed availability and feed nutritional composition, reproductive stage, fasting period, among others (23, 24). Processing methodologies, as salting and drying are incorrect or poorly executed, it may result in loss of protein or negatively affect its quality. However, in this study, all cod samples were processed equally, following Riberalves Good Manufacturing Practices. Therefore, differences observed between harvesting locations in the North Atlantic could be consequence of differences in: (1) reproductive stage at harvesting time; (2) feed availability or even in feed nutritional composition. On the other hand, differences between the Atlantic and Pacific species are probably due to distinct spawning periods, different feeding preferences/availability or even genetic dissimilarities. The differences observed between TP and TAA contents suggest that the amount of non-protein nitrogen is between 1.06 and 1.63 g/100 g.

### 4.2. Amino acid profile

A good protein source must present high digestibility, and contain adequate amounts of nutritionally essential or indispensable amino acids (IAA) such as: histidine, isoleucine, leucine, lysine, methionine, phenylalanine, threonine, tryptophan and valine, as well as those classified as conditionally indispensable amino acids (CIAA): tyrosine, glycine, arginine and proline, since they can become indispensable under specific physiological or pathological conditions, and sufficient amount of total amino acid nitrogen, which can be supplied by the dispensable amino acids (DAA), as alanine, aspartic acid, glutamic acid, hydroxyproline, ornithine and serine or even other sources of non-essential nitrogen (25).

The higher TP and TAA values in Atlantic cod, relatively to the Pacific cod, is in agreement with the total protein content referenced for the two species in the literature (26) and on the USDA database (27, 28). Differences between cod species in TAA and in the AA profiles may be dependent on differences in their genetic codes. Nevertheless, such assumption does not explain the differences observed between specimens obtained from different harvest locations within Atlantic, since the Atlantic cod appears to display low genetic variability throughout its range (29).

Seasonal variation in the fish muscle composition and differences observed between cod obtained from different harvest locations have been previously identified, and attributed to variability in feeding availability and to energy mobilization during the spawning period (30, 31). The Atlantic cod-spawning period varies between subpopulations, but most of them spawn from December to June (32). The samples used herein were harvested between January and April, a period that can include specimens in the pre- and post-spawning phase. Moreover, the winter is a period of low feed availability, which may determine some protein mobilization from the muscle tissue (26). The rate and extent of muscle protein mobilization should depend on feed availability and on energy demand to spawning. Therefore, differences observed between Icelandic and Norwegian cod in terms of AA partial sums and individual AA content suggest that they were caught in different physiological phases or that they were harvested from stocks with different feed availability.

Additionally, protein mobilization from the white muscle fibers has a stronger influence in sarcoplasmic proteins rather than the myofibrillar fraction (31). The distinct mobilization of protein from different fractions may also contribute to the differences observed in the AA profile between Icelandic and Norwegian cod, since different protein fractions comprise different AA profile.

The presence of hydroxyproline among the ten predominant AA in Pacific cod edible portion suggests that collagen is an important protein in this species, a condition that was not observed in the Atlantic cod. Our suggestion is sustained by the following data: (1) hydroxyproline is almost exclusive to collagen proteins, being present in insignificant amounts in other proteins (33); (2) the Pacific cod’s hydroxyproline content presented a superiority of 81.5% over the Atlantic cod; (3) the Pacific cod’s edible portion revealed a superiority over the Atlantic cod in the contents of proline and glycine (more 54.3 and 40.7%, respectively), which are two important amino acids in collagen proteins (25).

The identification of the LAA is an alternative approach to evaluate protein quality of most foods. Such assessment of LAA in cod’s edible portion shows that the Atlantic and Pacific cod species share 4 LAA, namely tryptophan (23–37%), the most limiting AA of all in the three cod varieties, followed by methionine (47–69%), valine (51–72%) and isoleucine (61–76%). Beyond, these 4 LAA, each origin presents an additional LAA, namely, histidine was a LAA in both the Pacific cod and Norwegian cod (92 and 85%), respectively, while lysine (91%) was a LAA for the Icelandic cod.

The LAA in cod’s edible portion is in accordance with the LAA in several foods (34). On the other hand, threonine has been identified as a common LAA in several protein sources, but is present in high concentration in cod’s edible portion.

Beyond the AAS, which assesses whether the protein in evaluation embraces individual IAA in an amount similar to a reference protein, the essential amino acid index (EAAI) evaluates the protein quality through the geometric mean value of all the IAA in relation to a reference protein (35). The comparison of cod species showed that the Atlantic cod had a significantly lower EAAI than the Pacific cod (*p* < 0.001; 88.23 versus 88.61). On the other hand, the comparison of the two origins in the Atlantic cod, showed that Norwegian cod displayed a significantly higher EAAI than the Icelandic cod (99.15 versus 77.32). The assessment of the EAAI allows the classification of the protein’s nutritional quality, accordingly: matching quality (EAAI ≥ 100%); high quality (between 95 and 100%); good quality (between 86 and 95%); useful quality (between 75 and 86%) and inadequate quality < 75% (36). Consequently, within the Atlantic cod, the Norwegian cod is classified as high quality, while the Icelandic cod attain the classification of useful quality, and the Pacific cod receives the good quality grade (36, 37).

### 4.3. Discriminatory ability of cod’ amino acid profile

The cod’s flesh amino acids with the highest discriminant power were the isoleucine, ornitine, total AA and arginine in root 1 and the ornitine, valine, alanine and arginine in root 2. Nonetheless, the discriminant effect between the Norwegian and Icelandic cod was not completely achieved. The AA composition of proteins is genetically encoded in the DNA, therefore, the results suggest genetic differences between cod species, but lack of genetic variability among the Atlantic cod. Such results are in agreement with the results showing low genetic variability in the Atlantic cod stocks (29).

## 5. Conclusion

The influence of cod’s specie on protein composition were observable in several variables. The AA profile was associated with 12 significant differences (*p* < 0.05), out of 19 individual AA, and such AA were accountable for 57.8–62.1% of TAA in cod’s edible portion. The Atlantic cod displayed higher TAA, total IAA, total CIAA, and total DAA, but exhibited lower IAA/TAA and IAA/DAA ratios than the Pacific cod. The Atlantic cod’s edible portion presented higher TP and TAA contents than the Pacific counterpart. Despite such differences, independently of the cod’s specie, its protein was classified as good quality and both display the same 5 LAA (histidine, isoleucine, methionine, tryptophan and valine). The presence of hydroxyproline among the ten predominant AA in Pacific cod edible portion suggests that collagen is an important protein in this species.

The harvesting location in the Atlantic cod had no influence on TP and TAA contents, and had a minor influence on cod’s protein quality. Still, Norwegian cod protein displayed higher TAA and IAA and higher IAA/TAA ratio. Independently of the harvesting location, both origins present 5 LAA and share 4 LAA. Regarding the EAAI, the Norwegian cod protein obtained a better score, being for that reason classified as high quality, while the Icelandic cod protein attain the classification of useful quality,

The third objective of this study was to evaluate AA profile discriminatory potential to classify cod samples. The results show that the AA profile has 100% accuracy in the separation of cod species, but was not globally efficient in the differentiation of the Norwegian from the Icelandic cod.

## Data availability statement

The raw data supporting the conclusions of this article will be made available by the authors, without undue reservation.

## Ethics statement

Ethical review and approval was not required for the animal study because the study included cod specimens that were legally harvested under regular fishing conditions.

## Author contributions

MQ was responsible for conception, design of the study, and wrote the first draft of the manuscript. CSp, AJ, and HG executed all analytical procedures. CSa and LR validated the analytical protocols, data curation, and organized the database. MQ and GP performed the statistical analysis. MN and LR wrote sections of the manuscript. CSa and MQ were answerable for funding acquisition. LR, MN, and MQ were accountable for project administration and supervision. All authors contributed to the article and approved the submitted version.

## Funding

This work was supported by FCT/MCTES through national funds, namely CIISA (UIDB/00276/2020) and AL4AnimalS (LA/P/0059/2020).

## Conflict of interest

The authors declare that the research was conducted in the absence of any commercial or financial relationships that could be construed as a potential conflict of interest.

## Publisher’s note

All claims expressed in this article are solely those of the authors and do not necessarily represent those of their affiliated organizations, or those of the publisher, the editors and the reviewers. Any product that may be evaluated in this article, or claim that may be made by its manufacturer, is not guaranteed or endorsed by the publisher.
